# Mayo Clinic VT calculator: A practical tool for accurate wide complex tachycardia differentiation

**DOI:** 10.1111/anec.13085

**Published:** 2023-09-05

**Authors:** Anthony H. Kashou, Sarah LoCoco, Matthew R. Gardner, Jocelyn Webb, Jacob C. Jentzer, Peter A. Noseworthy, Christopher V. DeSimone, Abhishek J. Deshmukh, Samuel J. Asirvatham, Adam M. May

**Affiliations:** ^1^ Department of Cardiovascular Medicine Mayo Clinic Rochester Minnesota USA; ^2^ Department of Medicine Washington University School of Medicine St. Louis Missouri USA; ^3^ Mayo Clinic Center for Digital Health Mayo Clinic Rochester Minnesota USA; ^4^ Cardiovascular Division Washington University School of Medicine St. Louis Missouri USA

**Keywords:** electrocardiogram, supraventricular tachycardia, ventricular tachycardia, wide complex tachycardia

## Abstract

The discrimination of ventricular tachycardia (VT) versus supraventricular wide complex tachycardia (SWCT) via 12‐lead electrocardiogram (ECG) is crucial for achieving appropriate, high‐quality, and cost‐effective care in patients presenting with wide QRS complex tachycardia (WCT). Decades of rigorous research have brought forth an expanding arsenal of applicable manual algorithm methods for differentiating WCTs. However, these algorithms are limited by their heavy reliance on the ECG interpreter for their proper execution. Herein, we introduce the Mayo Clinic ventricular tachycardia calculator (MC‐VTcalc) as a novel generalizable, accurate, and easy‐to‐use means to estimate VT probability independent of ECG interpreter competency. The MC‐VTcalc, through the use of web‐based and mobile device platforms, only requires the entry of computerized measurements (i.e., QRS duration, QRS axis, and T‐wave axis) that are routinely displayed on standard 12‐lead ECG recordings.

## Graphical abstract

Visual depiction of the web‐based platform interface for the Mayo Clinic ventricular tachycardia calculator is presented. The calculator allows users to input data from baseline and wide complex tachycardia ECGs in order to generate an estimate of VT probability. To use the Mayo Clinic ventricular tachycardia calculator, users are required to enter three key parameters: (i) QRS duration, (ii) QRS axis, and (iii) T‐wave axis, obtained from the displayed values of paired WCT and baseline ECG recordings. Upon entering these values, the calculator provides an automated estimation of VT probability, ranging from 0.000% to 99.999%.

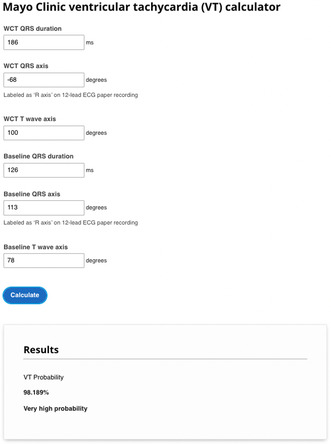

Among patients presenting with a regular wide QRS complex tachycardia (WCT), it is crucial to rapidly differentiate those with potentially life‐threatening ventricular tachycardia (VT) from those with supraventricular wide complex tachycardia (SWCT) who typically have a more favorable prognosis. Accurate and timely discrimination of VT and SWCT by way of 12‐lead electrocardiogram (ECG) interpretation is indispensable for appropriate, high‐quality, and cost‐effective management of patients who present with WCT. Yet, discriminating VT from SWCT continues to be a difficult task that has vexed clinicians for more than half of a century (Kashou et al., 2021; Kashou, Noseworthy, DeSimone, et al., 2020). Currently, clinicians must carefully inspect patients' 12‐lead ECG and assiduously apply one or more manual WCT differentiation criteria or algorithms to arrive at the correct VT or SWCT diagnosis (Brugada et al., 1991; Chen et al., 2019; Griffith et al., 1994; Jastrzebski et al., 2016; Kindwall et al., 1988; Moccetti et al., 2022; Pava et al., 2010; Vereckei et al., 2008; Wellens et al., 1978). While standard manual ECG interpretation methods have proven their diagnostic value in research settings, each is naturally vulnerable to their improper application or lack of use in “real life” clinical practice. This is an obvious shortcoming, especially when patients with WCT are cared for by medical providers who ordinarily do not possess a practiced proficiency in differentiating WCTs (e.g., medical trainees (May et al., 2018)). Furthermore, all existing manual WCT discrimination algorithms are inherently constrained by the inclusion of subjective or imprecise interpretation criteria, which can compromise the diagnostic sensitivity or specificity for VT and ultimately impede diagnostic performance in real‐world scenarios.

Recently, several novel WCT differentiation models (Kashou, DeSimone, Deshmukh, et al., 2020; Kashou, DeSimone, Hodge, et al., 2020; Kashou, LoCoco, McGill, et al., 2022; Kashou, LoCoco, Shaikh, et al., 2022; May et al., 2019a, 2019b, 2020) have been devised to counter the lingering difficulties inherent among manually operated WCT differentiation approaches. By design, automated WCT differentiation approaches exclusively use readily available computerized measurement data from paired WCT and baseline ECGs to deliver clinicians an automatic estimation of VT probability (0.000–99.999%). One such method—the VT Prediction Model (Kashou, DeSimone, Hodge, et al., 2020; May et al., 2020)—deliberately makes use of automated ECG measurements (i.e., QRS duration, QRS axis, and T‐wave axis) routinely displayed on WCT and baseline ECG recordings (Figure 1). As such, the VT Prediction Model transforms computerized measurements into diagnostic parameters (i.e., QRS duration, QRS duration change, QRS axis change, and T‐wave axis change) leveraged by logistic regression modeling, which delivers an impartial estimation of VT probability. In a two part study, we developed and validated the VT Prediction Model (May et al., 2020). In Part 1, the VT Prediction Model was derived from a dataset of 601 paired WCT (273 VT, 328 SWCT) and subsequent baseline ECGs, from 421 patients, using logistic regression modeling. In Part 2, the performance of the VT Prediction Model was evaluated using a separate validation cohort of 241 paired WCT (97 VT, 144 SWCT) and baseline ECGs from 177 patients. The overall diagnostic performance was excellent (area under the receiver operator curve [AUC] 0.900). In addition, the VT Prediction Model successfully generated a continuum of VT probabilities with favorable diagnostic performance indices across various VT probability thresholds. More recently, a subsequent prospective analysis (Kashou, Noseworthy, Jentzer, et al., 2022), which evaluated the VT Prediction Model's application by physician trainees, showed that it favorably improved ECG interpretation accuracy and interpreter confidence for discriminating WCTs.

**FIGURE 1:**
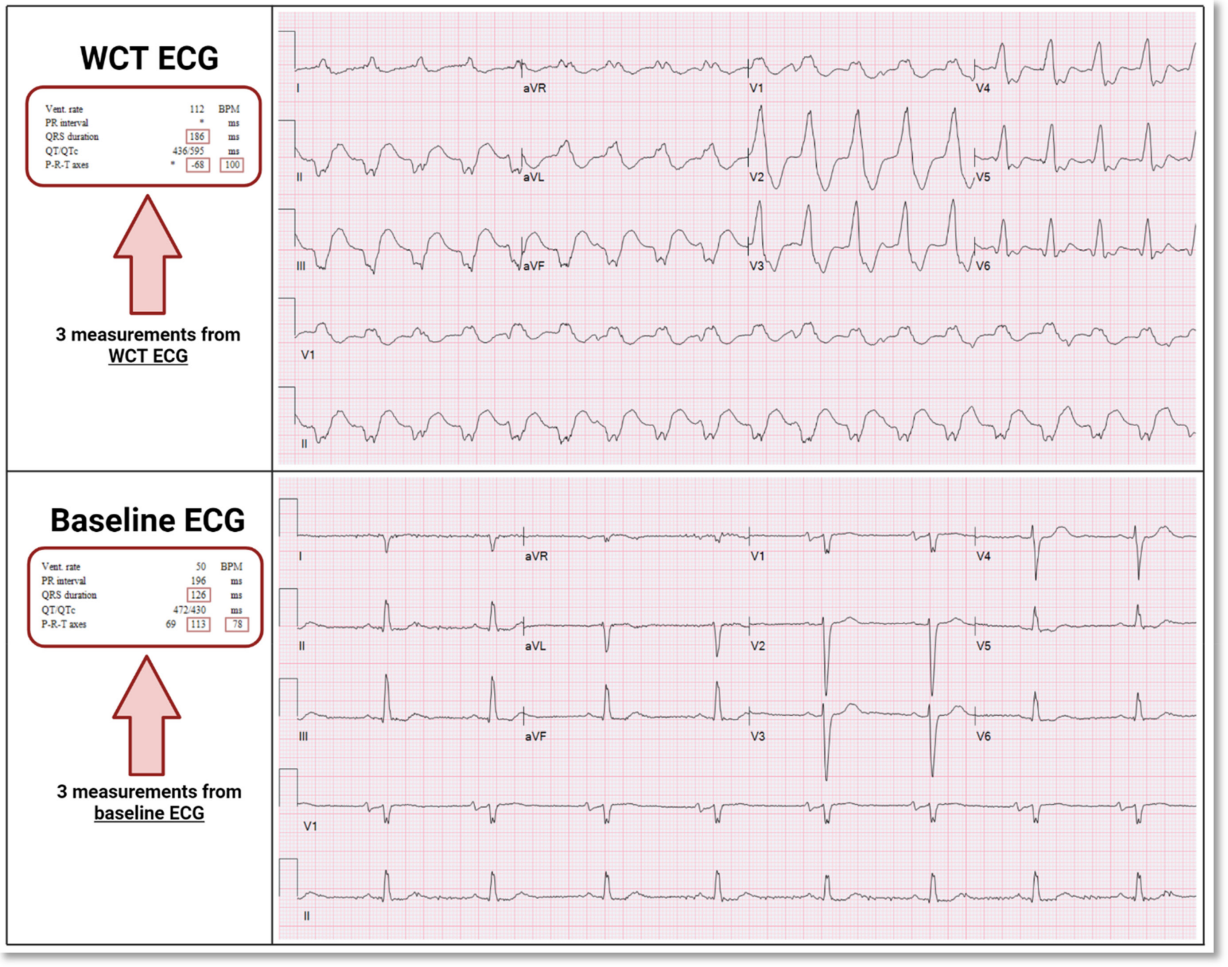
Measurements of WCT and baseline ECG pairs. Standard 12‐lead ECGs recordings of a WCT (confirmed VT) and baseline ECG pair. Computerized ECG measurements (QRS duration, R axis [i.e., QRS axis], and T axis [i.e., T‐wave axis]) from the WCT and baseline ECG that need to be manually entered into the MC‐VTcalc (shown in Figure 2) are highlighted. Figure created with BioRender.com. ECG, electrocardiogram; MC‐VTcalc, Mayo Clinic ventricular tachycardia calculator; VT, ventricular tachycardia; WCT, wide QRS tachycardia.

Given the well‐known challenges of executing manual WCT differentiation methods in clinical practice, we sought to offer a practical diagnostic solution that does not require manual ECG interpretation. By using the same mathematical expression of the VT Prediction Model (Kashou, DeSimone, Hodge, et al., 2020; May et al., 2020), we created a tangible and simple‐to‐use tool to help healthcare providers differentiate WCTs accurately—the Mayo Clinic Ventricular Tachycardia calculator (MC‐VTcalc) (Figure 2) (Mayo Clinic VT Calculator website). The MC‐VTcalc, through the use of web‐based and mobile device platforms, merely requires the manual entry of computerized measurements (i.e., QRS duration, QRS axis, and T‐wave axis) routinely displayed on WCT and baseline ECG recordings. To operate MC‐VTcalc, both WCT and baseline ECG measurement data must be entered. Once the data are entered, the MC‐VTcalc generates an estimation of VT probability ranging from 0.000 to 99.999%. The internal logic of the MC‐VTcalc is summarized in Figures S1 and S2. Once a VT probability is generated, it can integrated independently with other significant clinical factors, such as a history of myocardial infarction, and/or diagnoses obtained through alternative WCT differentiation methods (e.g., Brugada algorithm (Brugada et al., 1991), Vereckei aVR algorithm (Vereckei et al., 2008), or VT score (Jastrzebski et al., 2016)). Alternatively, the MC‐VTcalc has the potential to serve as an integrated algorithm that is automatically applied by computerized ECG interpretation software (previously unavailable). In future versions of the MC‐VTcalc, we plan to integrate additional readily accessible clinical data (such as the patient's history of structural heart disease) to further improve the overall diagnostic accuracy.

**FIGURE 2:**
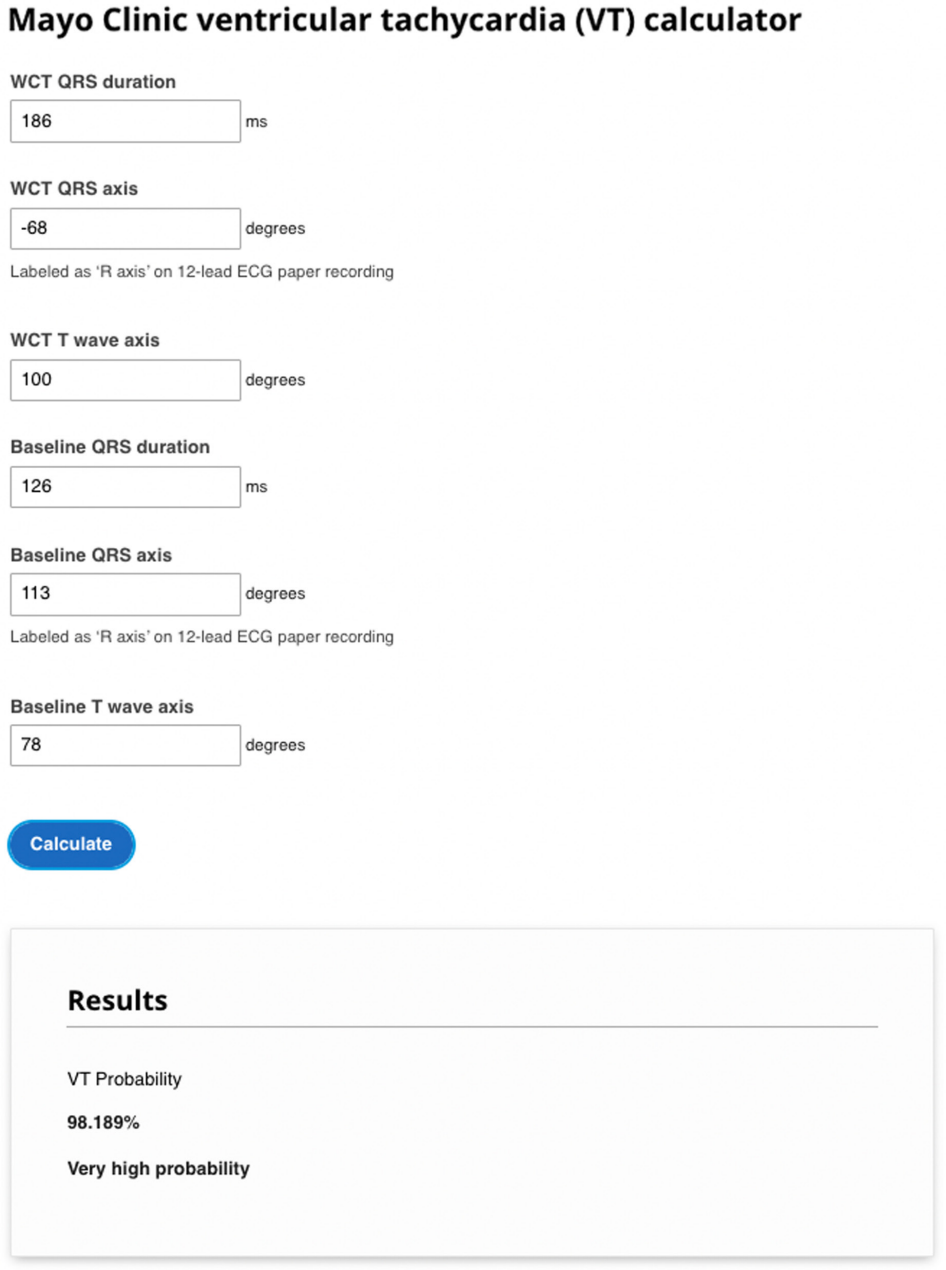
Mayo Clinic VT calculator. Visual depiction of the web‐based platform interface for the MC‐VTcalc. Data inputs from the ECG pair shown in Figure 1 (depicting a confirmed VT) were used for VT probability (%) calculation. Users of the MC‐VTcalc must enter (i) QRS duration, (ii) QRS axis, and (iii) T‐wave axis from the displayed values of the WCT and baseline ECG recordings. Once values are entered, an automated estimation of VT probability (0.000% ‐ 99.999%) is displayed. Of note, the baseline ECG may be the nearest ECG in time (before or after) from the WCT. At the time of this publication, the MC‐VTcalc may be accessed at Mayo Clinic VT Calculator website. MC‐VTcalc, Mayo Clinic ventricular tachycardia calculator; VT, ventricular tachycardia; WCT, wide complex tachycardia.

While decades of rigorous research have brought forth an expanding arsenal of manual methods to differentiate WCTs (Brugada et al., 1991; Chen et al., 2019; Griffith et al., 1994; Jastrzebski et al., 2016; Kindwall et al., 1988; Moccetti et al., 2022; Pava et al., 2010; Vereckei et al., 2008; Wellens et al., 1978), only recent works (Kashou, DeSimone, Deshmukh, et al., 2020; Kashou, LoCoco, McGill, et al., 2022; Kashou, LoCoco, Shaikh, et al., 2022; May et al., 2019a, 2020) have attempted to escape the limitations generally conceded by manual ECG interpretation approaches. Herein, we have introduced the MC‐VTcalc as a novel, tangible, generalizable, and easy‐to‐use means to provide an unambiguous estimation of VT probability independent of ECG interpreter competency. Until the development and effective implementation of machine learning predictive analytics or advanced artificial intelligence technologies (such as deep convolutional neural networks) for this specific purpose (Quer et al., 2021), practical diagnostic tools like the MC‐VTcalc can act as a vital “bridge” between the present and the anticipated future. By continually improving, refining, and integrating similar WCT differentiation tools, we can provide earlier assistance to clinicians in addressing a longstanding and complex clinical issue.

## AUTHOR CONTRIBUTION

Adam May: Conceptualization, Writing ‐ Original Draft, Writing ‐ Review & Editing. Anthony Kashou: Writing ‐ Original Draft, Writing ‐ Review & Editing. All other authors: Writing ‐ Review & Editing.

## FUNDING INFORMATION

This work was supported by the Department of Cardiovascular Medicine at Mayo Clinic in Rochester, MN. The authors also acknowledge support by NIH T32 HL007111.

## CONFLICT OF INTEREST STATEMENT

Anthony Kashou, Peter Noseworthy, Christopher DeSimone, Abhishek Deshmukh, and Adam May are possible future financial beneficiaries of intellectual property discussed in the article. The remaining authors do not have relevant disclosures to report.

## Supporting information


Figures S1 and S2.

## Data Availability

The data that support the findings of this study are available upon reasonable request. Interested researchers may contact the corresponding author to inquire about access to the data. The data will be shared in accordance with relevant ethical and legal considerations, ensuring the privacy and confidentiality of the participants.
