# Supplementary material for: Mayo Clinic VT calculator: A practical tool for accurate wide complex tachycardia differentiation
Source: Ann Noninvasive Electrocardiol. 2023 Sep 5;28(6):e13085. doi: 10.1111/anec.13085 (PMC10646384; doi:10.1111/anec.13085)
Supplement: Supplementary file 1 — Figures S1 and S2. [file ANEC-28-e13085-s001.docx]

**
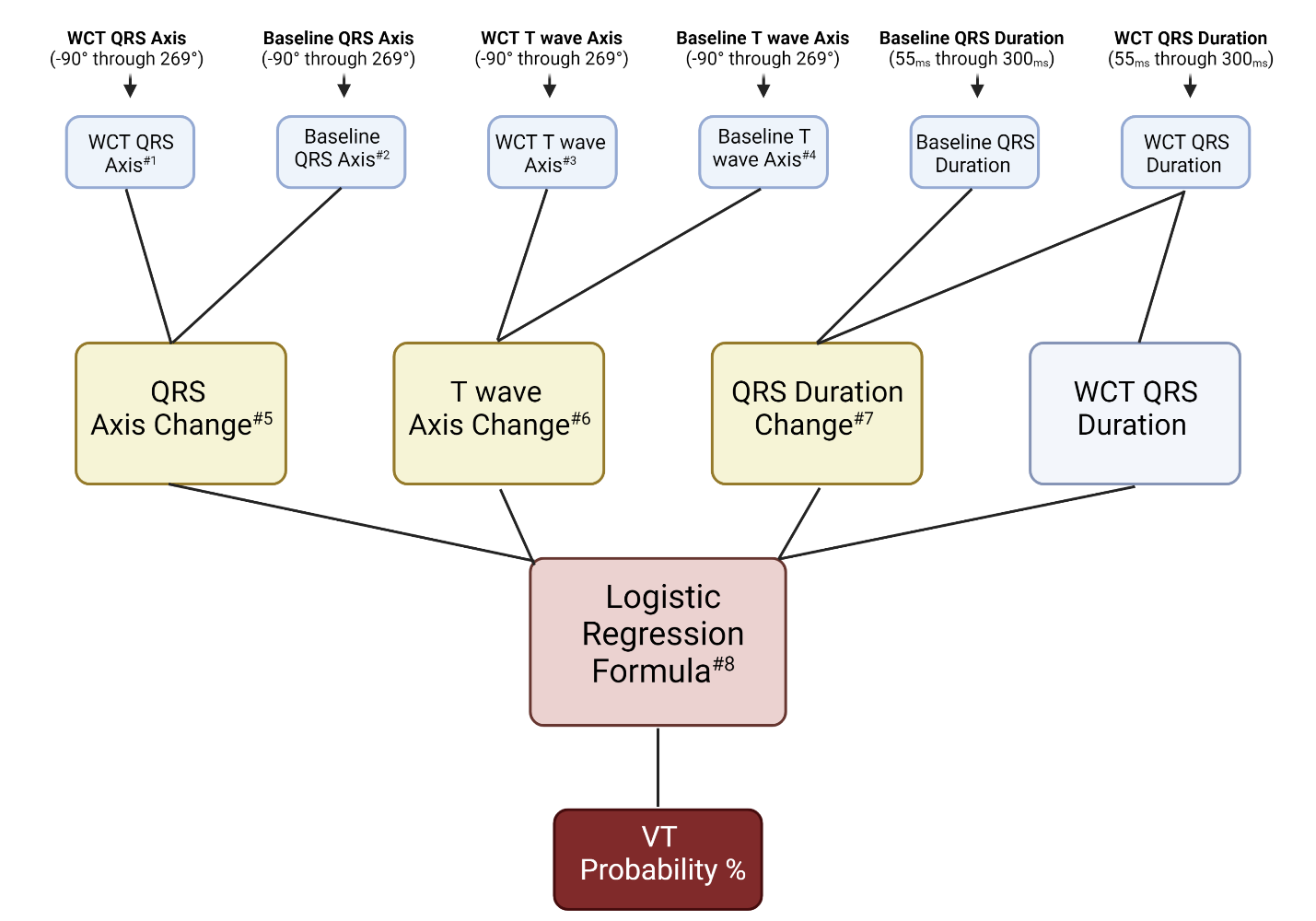
**

**Supplemental Figure 1: Mayo Clinic VT Calculator Internal Logic.** Flowchart is a visual depiction of the internal logic of the calculator leading to the output of VT probability (%). Logic equations are shown separately in **Supplemental Figure 2**. Figure created with *BioRender.com*.

*Abbreviations: WCT, wide complex tachycardia*; *VT, ventricular tachycardia.*

**
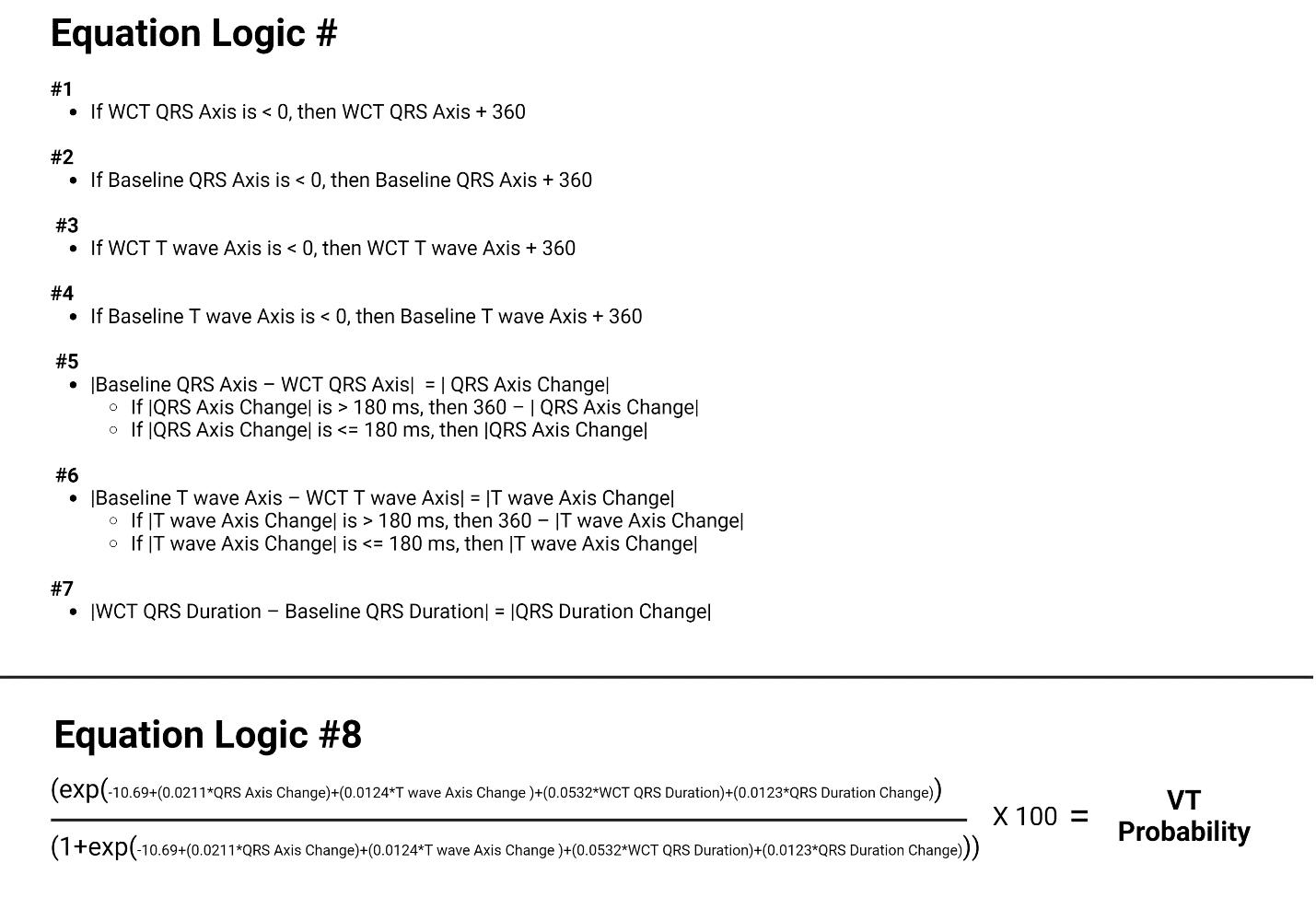
**

**Supplemental Figure 2: Equation Logic for Mayo Clinic VT calculator.** Please refer to **Supplemental Figure 1** to see the locations of the equations that reside in the internal logic of the Mayo Clinic VT calculator. Figure created with *BioRender.com*.

*Abbreviations: WCT, wide complex tachycardia*; *VT, ventricular tachycardia.*
